# Monomania Induced through Imitation

**Published:** 1844-12

**Authors:** 


*Monomania Induced through Imitation.*—Instinctive monomania, transmitted by imitation, although of an origin more mysterious and more difficult to explain, still shows itself in a more marked manner, and whenever it exhibits itself cannot be mistaken.

This branch of my subject can only be established by facts, but which in the present state of our knowledge cannot be satisfactorily explained. The following are examples:

An individual of a melancholic turn of mind, witnessed the execution of a criminal. This sight induced such violent exertion, that he was seized with a most vehement desire to kill some one, while at the same time he retained the most vivid fear of committing it. He stated his miserable condition in great anguish, beat his head, wrung his hands, and cried to his friends to save him. It would seem that in this instance the sight of blood produced a result similar to the impulse which actuates the insane in their periodical attacks of fury.

A child about seven years old strangled its younger brother. His parents caught him in the act. They asked him the cause. He replied weeping, that he was only imitating the Devil whom he had seen strangling Punch.

After the double murder (of children unknown to him) by Papavoine, a female of high rank visiting the place where the homicide had been committed, was seized on the instant with a desire to commit murder. The influence of printing an account of acts of this description in propagating this frightful instinct, is well known. Many instances occurred after the murder committed by Henrietta Cornier. Many mothers declared themselves impelled to destroy their children, and ask for aid to prevent the crime. At Amiens, a mother recently confined having heard of that person, was thus seized. She struggled against the impulse as long as possible, until at last fearing the result, she confessed her condition to her husband, who caused her to be secluded. In another instance, ten days after Cornier was sentenced, a mother strangled her child by pressing her arm around its neck.

*Suicidal Monomania transmitted by Imitation.*—A disordered state of the mental faculties not only impells men to the destruction of others, but also to their own. Strange passion, this of suicide! And yet, it is contagious, it is epidemic, it is very frequently the result of imitation. Examples crowd upon us from the earliest antiquity. Thus with the females of Miletus, the epidemic could alone be arrested by ordering that all the self-destroyers should be exposed publicly, naked, and with the cord around their necks.

After the invasion by the Spaniards, the Peruvians and Mexicans killed themselves in such numbers, that according to the historian, more fell by their own hands than by the weapons of their enemies.

In a small village in France in 1813, a female hung herself. Her example was soon followed by several others, and it needed exhortations of the Parish cure to check it.

M. Esquirol asks whether this epidemic appearance of suicide depends on an unknown condition of the atmosphere, on the imitation which propagates it, on political disasters in a country, or on a dominant opinion favorable to suicide. Doubtless it may originate from various causes, but in considering these, we should distinguish between predisposing and determining causes. It is evident, that in suicidal monomania, there exists a variety of predispositions. Temperature, the seasons, menstruation, pregnancy, political troubles, affections of the heart, all have induced it. But in epidemic monomania, although these single or united may operate in causing a propensity—yet the *determining* cause is almost invariably imitation. It is after the first case that these complex elements enter into fermentation and the epidemic bursts out.

Imitative suicide affects generally a most singular fidelity in the repetition of the act which it copies. This extends not only to the choice of the same means, but often of the same place, at the same age, and with the most exact resemblance of the previous scene.

A man (says Voltaire,) of a serious profession, middle age, very regular habits, and in comfortable circumstances, destroyed himself in October, 1769, and in a letter to the council of his native city, left the following apology: '*My father and my brother each killed themselves at the same age that I now am.*

Under the empire, a soldier destroyed himself in a sentry box. Several others repaired to the same, where they killed themselves. The sentry box was burnt and the epidemic ceased. Again, while Serrurier was Governor, an invalid hung himself on a door. In fifteen days, twelve invalids hung themselves on the same. Sabatier advised that the door should be walled up, which was done, and no new cases occurred.

*Other Monomanias transmitted by Imitation.*—If we allow that monomania may be transmitted in the preceding instances, it necessarily follows that it may be possible in other forms. Thus, it seems very evident to me that in many cases of incendiary monomania, as in England and Normandy, this may with probability be assigned as the cause.

Still, when monomania affects a number of persons at once, it must not invariably be attributed to a sympathetic imitation. This remark applies particularly to those kinds of reasoning monomania, which originate in pride and ambition. They are less exposed to public observation, and hence being unknown, cannot be imitated. Thus I witnessed shortly after the return of the Burbons, five or six females, who in total ignorance of each other's delusion, respectively imagined themselves to be the daughters of Marie Antoinette. Here could have been no imitation, since the public prints had given no account of them. At the same time also, we had many pretended dauphins, but as the papers noticed some of the cases, it is quite possible that several may have been the result of imitation. We may with greater certainty ascribe to imitation the extravagance committed by a number of young men in the city of Leipsic, who on the first representation of

Schiller's Robbers, abandoned their parents and repaired to a forest in order to form a band of brigands. We can readily explain the delusion in this case, and instead of referring it to a taste for robbery, assign the more natural cause—the charms of a nomade, free and independent life, as pictured forth in the German Drama.—*Ibid.*

